# Inherently at risk: Low floral morphological variability predicts conservation risk

**DOI:** 10.1017/ext.2026.10021

**Published:** 2026-09-29

**Authors:** Raymond L. Tremblay, Alex Leon-Rodríguez, Jose Colon Miranda, Valeria Meléndez Hebib

**Affiliations:** 1 Biology, University of Puerto Rico Rio Piedras, Puerto Rico; 2 Biology, University of Puerto Rico at Humacao, Puerto Rico; 3 Biological Sciences, Northern Illinois University, USA; 4 USDA Children’s Nutrition Research Center, Department of Pediatrics, Baylor College of Medicine, USA

**Keywords:** intraspecific trait variation, floral morphology, conservation triage, BIC model selection

## Abstract

Extinction risk should, in theory, be shaped not only by extrinsic pressures but also by intrinsic biological traits, including intraspecific floral morphological variability, which may influence ecological flexibility and pollination success. We evaluated whether reduced floral morphological dispersion, measured as species-level coefficients of variation (CVs) for seven floral dimensions, is associated with elevated conservation risk in Caribbean orchids. Class-weighted logistic regressions were fitted across multiple imputations, with exhaustive Bayesian Information Criterion selection across 127 candidate models. Single-trait models were favoured, petal-length CV best supported, followed by sepal-width and labellum-length. Sign-direction analyses confirmed that five perianth traits were predominantly negatively associated with risk (lower CV → higher risk), whereas column-length CV was robustly positive. Discrimination of Threatened from Not-Threatened species was modest but consistently above chance; under a precautionary threshold penalising missed detections, nearly all Threatened species were flagged. Because threat status was phylogenetically clustered (Fritz and Purvis D = 0.62), the full model set was refitted using phylogenetic logistic regression and taxonomic and phylogenetic mixed models; effect signs, magnitudes and rankings were preserved. Species at higher risk therefore tend to exhibit reduced intraspecific floral variation, and the model offers a biologically interpretable triage tool for prioritizing unassessed Caribbean orchids.

## Graphical abstract

**Figure:**
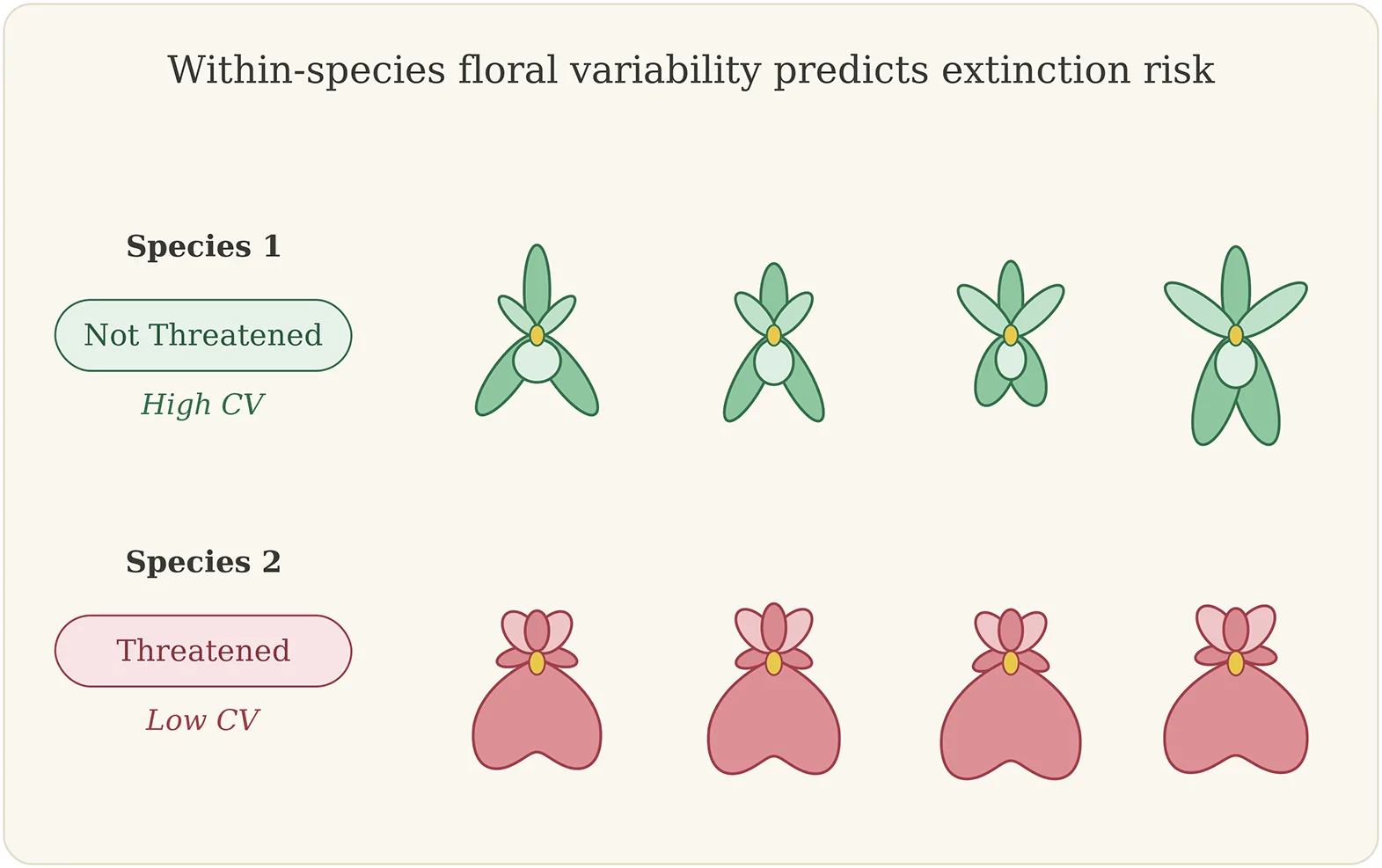

## Resumen

El riesgo de extinción depende no solo de presiones extrínsecas, sino también de rasgos intrínsecos como la variabilidad morfológica floral intraespecífica, que puede influir en la flexibilidad ecológica y la polinización. Evaluamos si una menor dispersión floral, medida como coeficientes de variación (CV) por especie en siete dimensiones florales, se asocia con mayor riesgo de conservación en orquídeas caribeñas. Se ajustaron regresiones logísticas ponderadas por clases sobre imputaciones múltiples, con selección exhaustiva de 127 modelos mediante el Criterio de Información Bayesiano. Se favorecieron modelos de un solo rasgo: el CV de la longitud del pétalo, seguido del ancho del sépalo y la longitud del labelo. Cinco rasgos del perianto se asociaron negativamente con el riesgo (menor CV → mayor riesgo), mientras que el CV de la longitud de la columna fue positivo. La discriminación fue modesta pero superior al azar; bajo un umbral precautorio se identificaron casi todas las especies amenazadas. El estado de amenaza mostró agrupamiento filogenético (D = 0.62); al reajustar los modelos con métodos filogenéticos y mixtos se conservaron signo, magnitud y orden. Las especies con mayor riesgo presentan menor variación floral intraespecífica, ofreciendo una herramienta de triaje interpretable para priorizar orquídeas caribeñas no evaluadas.

## Impact statement

Understanding why some species are more vulnerable to extinction than others is a central challenge in conservation biology. Most comparative studies use average trait values such as body size, range size and reproductive rate to predict which species face the greatest risk. Yet theory predicts that variation within a species, not just its average, should determine how well populations cope with environmental change, shifting interactions, and habitat disruption. We provide empirical evidence that reduced within-species variability in floral morphology is consistently associated with higher conservation risk. Species whose floral traits are more uniform are more often threatened than species with greater individual-to-individual variation. Such uniformity is consistent with the expectation that uniform populations hold fewer phenotypic variants able to maintain function under changing conditions, leaving a narrower margin for persistence when environments or interactions shift. Importantly, the consequences of variability depend on trait function: greater variation in structures involved in pollinator attraction may broaden ecological compatibility, whereas variation in structures requiring precise mechanical alignment for reproduction is associated with higher risk. Consequently, species may be *inherently at risk* through their own biology, independent of anthropogenic impact. Our framework converts morphometric measurements available in published floras into a continuous risk score, requiring no demographic surveys, habitat modelling or fieldwork. When calibrated to minimise missed detections, it correctly flags nearly all known Threatened species for priority assessment. We develop and test it in the Caribbean orchid flora, a hotspot of endemism and threat recently described in full by a single authority, which gives the measurements a uniform basis and minimises the variation that arises when data are compiled from many sources. Because flora-based morphometric data exist for many taxonomic groups worldwide, this offers a scalable, low-cost pathway for conservation triage in data-poor, biodiversity-rich regions where formal risk assessments remain incomplete.

## 1. Introduction

Persistence is the exception rather than the rule in evolutionary history: over 99% of all lineages that have ever existed are now extinct (Barnosky et al., 2011). Contemporary losses are driven primarily by anthropogenic pressures, yet whether a given lineage fails under those stresses may depend not only on the severity of external threats but also on intrinsic biological traits that modulate the taxonomic lineage’s exposure, sensitivity, and capacity to respond. Whether species carry trait syndromes that consistently elevate conservation risk, independently of the proximate cause of decline, remains an open question.

A substantial comparative literature has linked mean trait values to threat status. Elevated risk is repeatedly associated with small geographic range and narrow habitat breadth (Manne et al., 1999; Purvis et al., 2000; Slatyer et al., 2013), small population size and low abundance (Pimm et al., 1988; Lande, 1993; Brook et al., 2008), large body size (Cardillo et al., 2005; Ripple et al., 2017), slow life histories (Owens and Bennett, 2000; Cardillo et al., 2008), limited dispersal (Schloss et al., 2012) and low genetic diversity (Spielman et al., 2004; Frankham, 2005). A growing body of work suggests that low morphological or phenotypic variation is associated with increased extinction risk (Chevin et al., 2010; González-Suárez and Revilla, 2013), and recent meta-analyses have demonstrated that intraspecific effects on ecological processes can rival interspecific ones (Des et al., 2018), yet the emphasis in comparative risk assessment has remained largely on mean trait values rather than on the distribution of variation within species.

Intraspecific trait variation (ITV) is not measurement noise but a functional attribute of populations. ITV (assuming genetic basis) broadens realised niche space and provides raw material for phenotypic plasticity and adaptive change (Bolnick et al., 2011), a classic example being the rapid spread of industrial melanism in moths under environmental change (Kettlewell, 1955). At the population level, response diversity among individuals can sustain demographic performance during environmental downturns even without underlying strong genetic change, by ensuring that some individuals remain functionally matched to prevailing ecological conditions (Bolnick et al., 2011; Violle et al., 2012). Reduced ITV narrows compatibility with fluctuating environments and constrains both plastic and genetic routes to persistence (Chevin et al., 2010; Westerband et al., 2021). ITV can also permit short-term phenotypic adjustment near-range margins and sustain population performance under environmental variability (Christmas et al., 2016; Eriksson and Rafajlovic, 2022).

Orchids offer a particularly tractable system for examining this question. Floral morphology is a primary target of pollinator-mediated selection, as both pollinator access and reproductive efficiency frequently depends on tight structural coupling of some floral display traits and the mechanics of pollen transfer (Darwin, 1877; Nilsson, 1988; Johnson and Steiner, 1997; Schiestl and Cozzolino, 2008; Moré et al., 2012; Ackerman et al. 2023). Floral characters have nonetheless often been overlooked in broad comparative and functional-trait studies, despite their likely functional importance (Plos et al., 2026). Because most orchid species interact with few, often highly specialised and unique pollinators (Tremblay, 1992; Ackerman et al. 2023), even modest among-individual variation in floral geometry can determine whether a given flower is successfully pollinated or pollen is removed. The distribution of trait values within a population, rather than its central tendency, is therefore the ecologically relevant quantity. Populations with narrower morphological dispersion may interact with fewer pollinator phenotypes, experience more synchronised reproductive failure under unfavourable conditions, and possess less capacity for short-term phenotypic adjustment. The Caribbean orchid flora provides an unusually complete empirical substrate for this question: it comprises approximately 568 species documented in a single comprehensive regional treatment (Ackerman, 2014), with herbarium-based morphometric data to estimate within-species trait dispersion across a substantial fraction of the flora.

In practice, conservation prioritisation rarely proceeds from complete information. A large fraction of Caribbean orchids has never received a formal International Union for Conservation of Nature (IUCN) assessment, and assessment itself is constrained by expert time, funding, field access and the availability of scientists with the taxonomic expertise and interest needed to study particular groups. As a result, decisions about which species to evaluate first are shaped as much by interest, expertise, cost, feasibility and institutional opportunity as by biology. A transparent screen computable from data already held in published floras, requiring no new fieldwork, could help order this assessment queue by flagging candidate species for expedited evaluation. Such a screen is best understood as one input among many rather than a determination of threat status: it does not incorporate the economic, logistical and political considerations that ultimately govern conservation action, and is intended to complement, not replace, formal assessment. Because the cost of overlooking a declining species is high relative to that of an unnecessary assessment, even a modestly accurate screen that errs towards flagging can be operationally useful.

It was hypothesised that reduced within-species variability in floral morphology is associated with elevated conservation risk in Caribbean orchids. The underlying rationale is that lower phenotypic variability reflects diminished ecological flexibility and a reduced capacity to buffer environmental change, such that lineages expressing narrower morphological ranges are expected to be more vulnerable to decline. Accordingly, it was predicted that species with lower coefficients of variation (CVs) across perianth dimensions would exhibit a higher modelled probability of being classified as Threatened. The mechanistic drivers of any such pattern were not evaluated here; the present study was intended to establish whether the pattern is detectable and whether it carries practical value for triaging unassessed taxa.

Here we ask whether reduced within-species floral variability, quantified as small-sample-corrected CVs for seven floral dimensions, predicts a higher probability of conservation risk in Caribbean orchids. We focus on floral trait dispersion rather than mean values, apply complete-subset information-theoretic model selection and evaluate predictive performance across Monte Carlo cross-validation (MCCV) folds. Our goal is to determine whether specific floral traits show consistently reduced variability in species at elevated extinction risk.

## 2. Materials and methods

### 2.1. Data assembly

We assembled species-level floral measurements for the Caribbean orchid flora from Ackerman (2014), extracting sepal length and width, petal length and width, labellum length and width and column length (in mm) for each species. When only ranges were reported, we estimated the mean as the mid-range (min + max) / 2 and the standard deviation as one-quarter of the range, a classical range-based estimator (Hozo et al., 2005) assuming normal distribution of traits. The CV was computed using the unbiased small-sample correction (Sokal and Rohlf, 1995), which reduces downward bias at small sample sizes. The range-based estimator assumes approximate within-species normality; because the resulting among-species CV distributions are right-skewed, we relied on rank-based and class-weighted methods that do not assume normality in the downstream analyses.

### 2.2. Conservation status

IUCN categories were collapsed to a binary outcome: Threatened (Extinct, Extinct in the Wild, Critically Endangered, Endangered, Vulnerable, and Near Threatened) versus Not Threatened (Least Concern). This binary contrast was adopted because the finer risk categories are too unevenly and sparsely populated at this sample size to support a reliable ordinal or multinomial model: among the assessed Caribbean orchids, the Threatened species are heavily concentrated in the Endangered category (approximately 53 species), with Vulnerable represented by only five and the remaining categories (Critically Endangered, Near Threatened, Extinct in the Wild and Extinct) by fewer still. The two-class Threatened versus Not Threatened outcome also matches the screening aim of flagging species for assessment, and finer, ordinal resolution would require the larger cross-regional and cross-taxon datasets that we identify as a priority for future work. Species without a risk assessment (138 Data Deficient or Not Evaluated species) were excluded. The 138 excluded (Data Deficient or Not Evaluated) species amount to roughly one-third of those with compiled floral data (240 retained plus 138 excluded). Data Deficient and Not Evaluated taxa are, as in other groups, disproportionately likely to prove threatened once assessed, so the prevalence of threat in the full Caribbean flora is probably higher than in this assessed subset; restricting the analysis to assessed species is expected to be conservative for the trait–risk association rather than to inflate it, and these unassessed species are precisely those the screen developed here is intended to prioritise. The final dataset comprised 240 Caribbean orchid species, of which 66 (27.5%) were classified as Threatened. These species span 80 genera across the Greater Antilles and associated islands, and approximately one-third are single-island endemics.

### 2.3. Statistical model and model selection

We built a binary classification pipeline to predict Threatened status from the seven floral CVs. Data were partitioned into a stratified 70/30 training–test split (train *n* ≈ 170; test *n* ≈ 73) preserving class prevalence (Kuhn and Silge, 2023). Missing predictors were handled with Multiple Imputation by Chained Equations (MICE) using predictive mean matching (Rubin, 1987; van Buuren and Groothuis-Oudshoorn, 2011; van Buuren, 2018), generating m = 100 imputations separately for training and test sets (maxit = 20 iterations per chain). Missingness ranged from 13 to 54% across traits; a preliminary sensitivity analysis at m = 30 confirmed identical model rankings and directional conclusions, validating the choice of m = 100. Missingness arose chiefly from source reporting (for some species the flora reports a single measurement rather than a range, precluding a within-species CV)) and was higher among Threatened species for several traits, a form of outcome-related missingness (Dumelle et al., 2025). Because the imputation model excludes the outcome to avoid target leakage in the predictive framework, this is expected to bias trait–risk associations conservatively towards the null. We confirmed robustness by re-imputing with the outcome included and by complete-case analysis: the direction of every focal-trait effect was preserved (odds ratios <1 in all cases) and effects were, if anything, slightly stronger when the outcome was included (Supplementary Tables S6–S7 and Supplementary Figure S5).

We enumerated all 127 non-empty subsets of the seven CV predictors, removed zero-variance predictors within each imputation, and filtered collinearity at |r| ≥ 0.90. We additionally assessed collinearity among the seven CVs using the pooled Spearman rank correlation matrix (chosen because the CVs are strongly right-skewed and non-normal) and variance inflation factors (Supplementary Table S5 and Supplementary Figure S4). For each imputed training set and each predictor subset, we fitted a class-weighted logistic regression (inverse-frequency observation weights to address class imbalance). The Bayesian Information Criterion (BIC; Schwarz, 1978) was computed within each imputation and averaged across all m = 100 imputations to rank models; Akaike Information Criterion (AICc) was retained as a sensitivity criterion. The best model and candidate set were identified using a ΔBIC ≤ 2 threshold, corresponding to ‘substantial’ evidence against competing models (Burnham and Anderson, 2002). To interpret directionality, we fitted marginal univariate logistic models pooled across imputations for each trait and summarised BIC-weighted sign direction across the full 127-model candidate space. A comparison of models fitted on raw and log-transformed CVs showed negligible AIC and BIC differences (ΔBIC <0.05) and spline fits confirmed linearity, so untransformed CVs were retained.

We combined regression results from the 100 imputed datasets using Rubin’s rules (Rubin, 1987; Barnard and Rubin, 1999), which incorporates between-imputation uncertainty into pooled estimates. The resulting odds ratios and 95% confidence intervals reflect this uncertainty; wider intervals are expected given 13–54% missingness.

To characterise the magnitude and direction of differences in CV between Threatened and Not Threatened species, we computed Cliff’s δ for each floral trait (Cliff, 1993). Cliff’s δ is a non-parametric effect-size measure (range −1 to +1) quantifying the probability that a value from one group exceeds one from the other; values of ±0.147, ±0.330, and ±0.474 correspond to small, medium, and large effects, respectively (Romano et al., 2006). Negative δ indicates lower CV in Threatened species, consistent with the hypothesis that reduced variability is associated with higher risk. Wilcoxon rank-sum tests were applied to assess statistical significance, and effect sizes were considered alongside *P*-values in light of modest sample sizes.

### 2.4. Phylogenetic non-independence

Because closely related species may share both floral morphology and conservation status, we assessed and controlled for phylogenetic non-independence. We obtained a phylogeny for the study species from the Open Tree of Life synthetic tree (Hinchliff et al., 2015) with the rotl package (Michonneau et al., 2016), matching 238 of 240 names; branch lengths were assigned by Grafen’s method (Grafen, 1989). Phylogenetic signal in threat status was quantified with the Fritz and Purvis D statistic for binary traits (Fritz and Purvis, 2010). We then repeated the full 127-model BIC selection using phylogenetic logistic regression (phyloglm, logistic logistic maximum penalised likelihood estimation, MPLE, Ho and Ané, 2014), and refitted the focal single-trait models under two mixed-model specifications: a class-weighted binomial generalised linear mixed model with a genus random intercept (Bates et al., 2015), and a phylogenetic binomial mixed model with a species-level random effect whose covariance is the phylogenetic covariance matrix (Ives and Helmus, 2011; Li et al., 2020). Coefficients were pooled across the m = 100 imputations with Rubin’s rules. Full procedures and results are reported in the Supplementary Material (Supplementary Tables S2–S4).

### 2.5. Geographic covariate

We further tested whether the floral signal is confounded by geographic range, the strongest known correlate of extinction risk. From the distribution statements in Ackerman (2014), we coded each species as a single-island endemic (recorded from a single island) or as more widespread (two or more islands or the adjacent mainland), and refitted the focal single-trait models with this rarity covariate, pooling across the m = 100 imputations with Rubin’s rules (Supplementary Table S8).

### 2.6. Performance evaluation: Monte Carlo cross-validation

To obtain stable performance estimates given the modest sample size (*n* = 240), we evaluated the best BIC model using MCCV (Arlot and Celisse, 2010) with N = 500 stratified 70/30 splits of the full dataset. We applied the pre-selected best BIC model to each fold rather than repeating model selection within folds. Predictions within each fold were averaged across m = 20 imputations (impute-once-and-resample approximation; justified because missingness is in predictors, not the outcome). We report mean and SD of the Receiver Operating Characteristic (ROC AUC) and Precision–Recall (PR AUC) areas under the curve (AUC; hereafter ROC AUC and PR AUC) across the 500 folds together with 95% percentile confidence intervals. Because the response is imbalanced (27.5% Threatened), PR AUC is the primary discrimination metric, given its superior sensitivity to minority-class performance in such settings (Saito and Rehmsmeier, 2015). The PR naive baseline equals the Threatened prevalence in the test fold (0.274 throughout). Fold-to-fold variance (SD across 500 folds) reflects performance stability; high variance is expected when few Threatened species per fold (≈14–20) mean a single misclassification disproportionately affects the metric.

We assessed whether the observed mean ROC AUC exceeds chance using a permutation test (Ojala and Garriga, 2010): outcome labels were shuffled B = 10,000 times across the full dataset, and mean MCCV AUC (20 folds per permutation) was computed each time to construct a null distribution. The permutation *P*-value is the proportion of null AUCs ≥ the observed mean AUC.

### 2.7. Triage thresholds

Recall (also termed sensitivity) is the proportion of truly Threatened species that the model correctly flags for assessment, representing the fraction of genuine conservation priorities that would not be missed in operational use. A recall of 1.0 indicates that no Threatened species is overlooked, whereas a recall of 0.5 implies that half are missed. In a conservation triage context, recall is the most critical performance metric because the cost of failing to identify a likely Threatened species is substantially higher than the cost of unnecessarily assessing a non-threatened one.

We evaluated three thresholding rules to translate the continuous prioritisation score into operational guidance: (i) Youden’s J (Youden, 1950), which maximises sensitivity plus specificity and treats false negatives and false positives as equally costly, is included only as a neutral, cost-agnostic reference because in conservation triage a missed Threatened species is far costlier than an unnecessary assessment; (ii) a PR max-F1 rule, which maximises the harmonic mean of precision and recall (constrained to specificity >0.10); and (iii) a cost-sensitive threshold minimising 5 ×  (false negatives) + 1 × FP (false positives), explicitly reflecting the asymmetric costs of missing a Threatened species relative to triggering an unnecessary assessment (Elkan, 2001). All threshold-dependent metrics are reported as mean ± SD across 500 MCCV folds. Importantly, we treat the model as a continuous prioritisation index rather than a binary classifier of threat status.

Precision is the proportion of species flagged as Threatened that are genuinely Threatened and quantifies the degree of unnecessary assessment generated by the model. F1 is the harmonic mean of precision and recall, providing a single summary of their trade-off; it reaches 1.0 only when both metrics are perfect and declines when either is poor. These metrics are reported for completeness and transparency, but operational emphasis is placed on recall-oriented thresholds consistent with conservation triage objectives.

### 2.8. Software and reproducibility

All analyses were conducted in R version 4.6.1 (R Core Team, 2026). Imputation used the *mice* package (van Buuren and Groothuis-Oudshoorn, 2011); preprocessing and performance metrics used tidymodels (Kuhn and Wickham, 2020; Kuhn and Silge, 2023); and figures were produced with ggplot2 (Wickham, 2016). Phylogenetic analyses used the ape (Paradis and Schliep, 2019), rotl (Michonneau et al., 2016), phylolm (Ho and Ané, 2014), phyr (Li et al., 2020), and caper (Orme et al., 2018) packages. Complete scripts, imputation caches (m = 100), MCCV fold results (N = 500), model tables and predictions are openly archived (see the Data Availability Statement).

## 3. Results

### 3.1. Trait variation by conservation status

Floral traits varied considerably in their absolute level of morphological dispersion across species. Median CV ranged from 0.278 (sepal width and petal width) to 0.663 (sepal length) among Not Threatened species and from 0.214 (petal width) to 0.807 (labellum length) among Threatened species (Table 1). This roughly threefold range in dispersion across traits reflects the scale-free nature of the CV. Traits that differ substantially in absolute size such as column length versus sepal length, for instance, are nonetheless directly comparable as measures of within-species variability. The broad dispersion in CV values across traits suggests that floral structures differ inherently in how morphologically variable they tend to be, independent of conservation status, and thus merits evaluating each trait separately rather than aggregating across the floral phenotype.

**Table 1.** Median within-species coefficient of variation (CV) and interquartile range (IQR) for floral characters of Threatened and Not Threatened Caribbean orchid species Table 1. long description.

Floral trait	CV not threatened	IQR	CV threatened	IQR	*P*	Cliff’s δ
Sepal width	0.278 (139)	0.185–0.445	0.207 (46)	0.160–0.372	0.056	−0.188
Sepal length	0.673 (155)	0.408–1.040	0.568 (55)	0.368–1.080	0.266	−0.101
Petal length	0.584 (143)	0.326–1.000	0.509 (42)	0.304–1.000	0.398	−0.086
Labellum length	0.569 (124)	0.280–1.120	0.687 (23)	0.269–1.060	0.858	−0.024
Petal width	0.248 (125)	0.120–0.489	0.214 (34)	0.133–0.403	0.906	−0.013
Labellum width	0.422 (92)	0.224–0.824	0.456 (18)	0.186–0.878	0.948	−0.010
Column length	0.384 (104)	0.202–0.652	0.456 (27)	0.174–0.709	0.948	+0.009

*Note:* Traits are ordered by Cliff’s δ from most negative to most positive to emphasise patterns most consistent with the hypothesis that reduced within-species variability is associated with higher conservation risk (H1). Negative δ values indicate lower CV in Threatened species (consistent with H1); positive δ values indicate higher CV in Threatened species. Wilcoxon rank-sum *P*-values are reported alongside effect sizes given modest sample sizes. Sample sizes in parentheses.

Across six of seven floral traits, Threatened species exhibited lower or similar median CV compared with Not Threatened species, broadly consistent with the hypothesis that reduced trait variability is associated with elevated risk (Table 1). Sepal width showed the strongest directional effect, with Threatened species exhibiting lower median CV (0.207 vs. 0.278; Cliff’s δ = −0.188; *P*-value = 0.056). Petal and sepal length showed smaller but consistent negative effects (δ = −0.086 and − 0.101, respectively). Labellum length showed negligible difference between groups (Not Threatened median CV = 0.569, Threatened = 0.687; *P*-value = 0.858; δ = −0.024). Column length was the only trait where Threatened species showed marginally higher CV, consistent with the mechanical precision requirements of orchid pollination, though the effect was negligible (Cliff’s δ = +0.009; *P*-value = 0.948). Although no trait reached *P*-value <0.05, sepal width approached significance (*P*-value = 0.056), consistent with limited power given the small Threatened sample (*n* = 18–55 per trait); effect sizes provide the primary basis for interpretation at this stage.

### 3.2. Model selection: A three-model candidate set

Of the seven floral traits examined, within-species variability in petal length emerged as the strongest and most consistent predictor of conservation risk, followed by sepal width and labellum length. These three traits formed the entire candidate model set within ΔBIC ≤2 (Table 2), each as single-predictor models, suggesting that the signal resides in individual display of individual traits rather than in combinations of traits as none of the trait combination models were parsimonious. Petal-length CV alone accounted for half the normalised BIC weight within the candidate set (wBIC = 0.203; wBIC_norm = 0.509), with sepal-width CV and labellum-length CV contributing the remainder. No other floral trait approached this level of support across the 127 models evaluated. Collinearity among the seven CVs was moderate (all pairwise Spearman |rs| ≤ 0.81; all variance inflation factors were below 5, whether computed from the fitted linear models (maximum 4.79) or from rank-transformed predictors (maximum 4.80); Supplementary Table S5 and Supplementary Figure S4), so no predictor was removed by the |r| ≥ 0.90 filter. Because the CVs nonetheless share moderate covariance, we interpret the consistent signal across perianth traits rather than an independent effect of any single trait.

**Table 2.** BIC candidate model set (ΔBIC ≤2), comprising three models selected from 127 candidate subset models evaluated across *m* = 100 imputed datasets Table 2. long description.

Rank	*n* Features	Predictors	BIC mean	ΔBIC	wBIC (wBIC_norm)
1	1	Petal-length CV	298.87	0.00	0.203 (0.509)
2	1	Sepal-width CV	300.21	1.34	0.104 (0.260)
3	1	Labellum-length CV	300.45	1.58	0.092 (0.231)

*Note: w*BIC denotes the raw BIC model weight, while *w*BICnorm denotes the BIC weight normalised within the ΔBIC ≤2 candidate set.

Variable inclusion probabilities across all 127 models confirmed this hierarchy: petal length (wBIC = 0.516) > sepal width (0.348) > labellum length (0.267) > column length (0.216) > labellum width (0.141) = sepal length (0.141) > petal width (0.131; Supplementary Figure S1). Petal-length CV carries more than twice the summed BIC weight of any other trait except sepal width, reflecting its consistent presence in well-supported models. Targeted a priori interaction models among the leading perianth traits did not outperform the corresponding single-trait models. For example, the petal-length CV × sepal-width CV interaction increased BIC by 8.1 units relative to the best single-trait model, and the interaction term was non-significant (OR = 1.16, *P*-value = 0.76). The remaining pairwise interactions showed similar patterns (ΔBIC 7.8–8.4; all interaction *P*-values >0.5; Supplementary Table S9). The single-trait hierarchy is therefore not an artefact of unmodelled interactions.

### 3.3. Sign direction and marginal odds ratios

For most perianth display traits, species with less within-species variability were more likely to be classified as Threatened. This pattern was strongest and most consistent for petal length, sepal width and labellum length, where the direction of association was negative in virtually all models evaluated (100%, 100%, and ~100% of BIC weight, respectively), and odds ratios confirmed that higher CV associates with lower risk (petal length OR = 0.597, 95% CI [0.349, 1.023], *P*-value = 0.060; labellum length OR = 0.633, 95% CI [0.352, 1.139], *P*-value = 0.126; sepal width OR = 0.326, 95% CI [0.077, 1.391], *P*-value = 0.129). Labellum width and sepal length showed weaker but predominantly negative patterns (78% and 57% of BIC weight respectively), suggesting a broadly consistent signal across the outer perianth variables (Table 3).

**Table 3.** BIC weighted sign direction and marginal univariate odds ratios (OR) for the association between within species floral coefficient of variation (CV) and Threatened status Table 3. long description.

Trait	Incl. wBIC	% Neg.	% Pos.	OR	95% CI	Direction
Sepal width	0.348	100	0	0.326	[0.077, 1.391]	↓ CV → ↑ risk
Petal length	0.516	100	0	0.597	[0.349, 1.023]	↓ CV → ↑ risk
Labellum length	0.267	~100	~0	0.633	[0.352, 1.139]	↓ CV → ↑ risk
Sepal length	0.141	57	43	0.690	[0.405, 1.174]	Mixed
Labellum width	0.141	78	22	0.757	[0.422, 1.360]	Mostly ↓
Petal width	0.131	18	82	0.809	[0.257, 2.540]	Mostly ↑
Column length	0.216	4	96	0.954	[0.417, 2.183]	↑ CV → ↑ risk

*Note:* ORs are pooled across *m* = 100 imputed datasets using Rubin’s rules and are sorted from smallest to largest to emphasise traits for which reduced CV is most strongly associated with higher conservation risk. *w*BIC denotes the summed BIC inclusion weight for each trait across all 127 candidate models; values are not constrained to sum to one because traits may co-occur within models and is the proportion of total model support that lies in models containing that trait. ORs are expressed per unit increase in CV. All 95% confidence intervals include 1.0.

Column length showed a robustly positive sign direction across multivariate models (positive in 96% of BIC-weighted models), consistent with the pollinator precision hypothesis which would predict that tight spatial alignment between column and pollinator is essential for pollen transfer, so excess dispersion in this structure likely desynchronises fit rather than broadening compatibility. Its marginal odds ratio was near unity (OR = 0.954, *P*-value = 0.91), as the positive association only becomes apparent when perianth trait variability is held constant. Petal width also showed a predominantly positive association (82% of BIC weight), though with low overall inclusion weight (wBIC = 0.131) and a wide confidence interval are basically not supporting of the main hypothesis. For all traits, confidence intervals include 1.0, reflecting in part the modest sample size and possibly the high missingness; the directional consistency across 127 models and two analytical frameworks constitutes the primary inferential result (Figure 1; Supplementary Figure S2).

**Figure 1.**
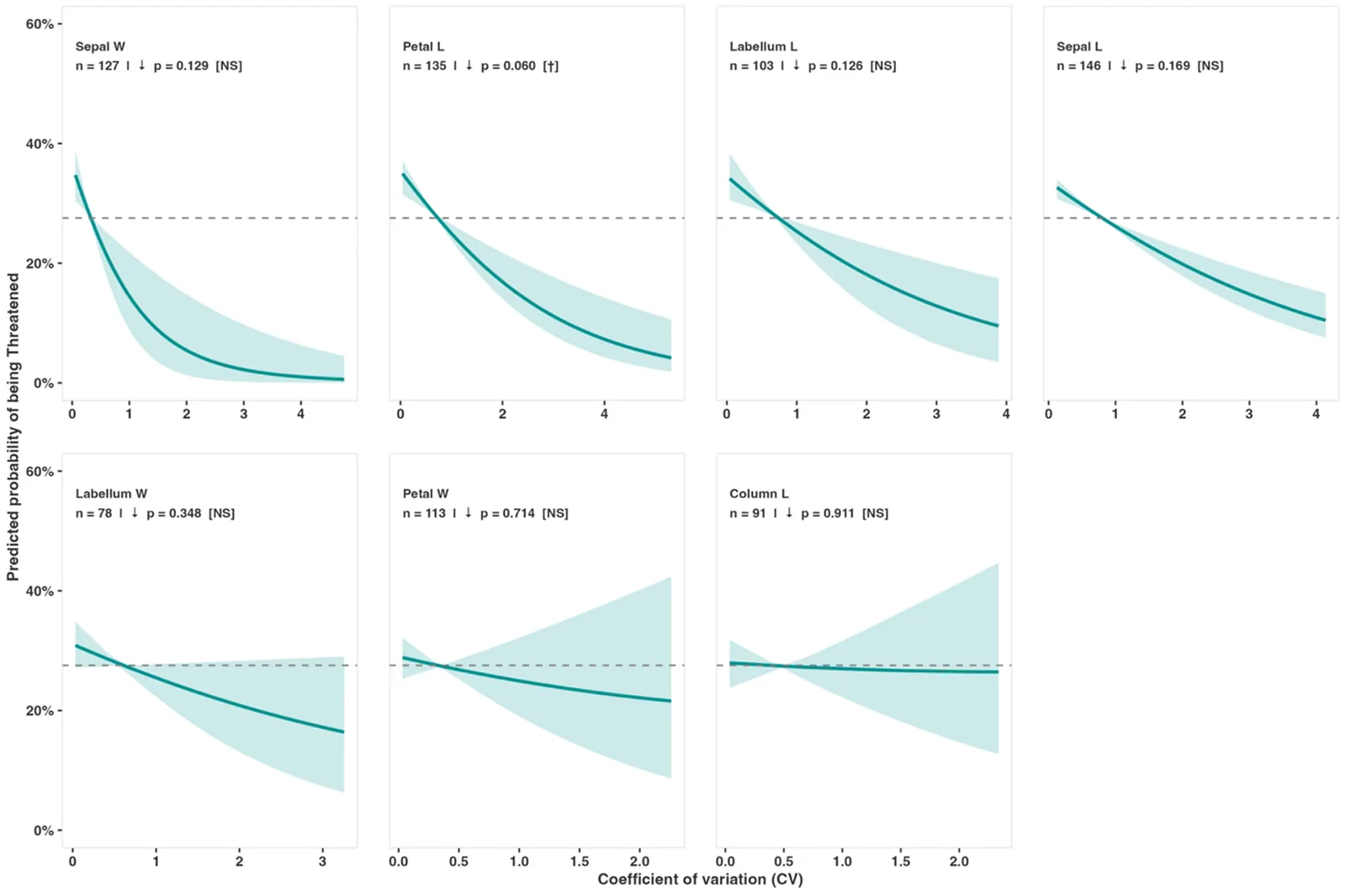
Marginal logistic relationships between within-species floral coefficient of variation (CV) and the predicted probability of Threatened status for seven focal floral traits in Caribbean orchids (training set, *n* = 170). Each panel displays the mean predicted probability (solid line) and the corresponding 95% multiple imputation envelope (shaded ribbon), derived from univariate logistic regressions fitted separately to each of the *m* = 100 MICE imputed datasets. The dashed horizontal line indicates the observed prevalence of Threatened species in the training set (27.5%). Panels are ordered left to right and top to bottom by increasing odds ratio, corresponding to the order reported in Table 3. Sample sizes (*n*) indicate the number of species with observed (non-imputed) values for each trait. Arrows denote the direction of the association (↓ lower CV associated with higher extinction risk; ↑ higher CV associated with higher extinction risk). *P*-values are pooled across imputations using Rubin’s rules; † NS *P*-value ≥ 0.10. Figure 1. long description.

### 3.4. Robustness to phylogenetic non-independence

Threat status was phylogenetically clustered rather than randomly distributed across the orchid phylogeny (Fritz and Purvis D = 0.62; departure from a random distribution *P*-value <0.001; departure from Brownian expectation *P*-value <0.001; Supplementary Table S2). We therefore repeated the analysis while modelling this structure. Under phylogenetic logistic regression, the ΔBIC ≤2 candidate set again comprised three single-trait perianth-CV models, yielding essentially the same set of supported models (Supplementary Table S3). The direction of every perianth trait remained negative, and column-length CV remained positive in multi-trait models. Coefficient estimates were essentially unchanged across the ordinary, phylogenetic, genus-random-effect and phylogenetic-random-effect frameworks (Supplementary Table S4); under the phylogenetic regression, the perianth effects were, if anything, marginally stronger (sepal-width CV β = −1.96, *P*-value = 0.054; petal-length CV β = −0.74, *P*-value = 0.082). Two independent estimates of residual phylogenetic correlation, the phyloglm α (≈ 54, at its upper bound) and the phylogenetic random-effect variance (≈ 0), both indicated negligible phylogenetic autocorrelation in risk after accounting for floral CV. The trait–risk association is, therefore, weak but robust and is unlikely to be an artefact of phylogenetic non-independence.

### 3.5. Geographic range and floral variability

Single-island endemics were far more likely to be Threatened than more widespread species (72% versus 7% among the 227 species with distribution data). Adding single-island endemism to the focal models confirmed it as a strong predictor of risk (odds ratio 23 to 33; *P*-value <0.001), yet the perianth-CV effects were not attenuated: petal-length (OR = 0.33, *P*-value = 0.017), sepal-width (OR = 0.39, *P*-value = 0.042) and labellum-length CV (OR = 0.44, *P*-value = 0.021) all became significant once island occupancy was controlled, whereas column-length CV remained near unity (Supplementary Table S8). Reduced perianth variability therefore predicts conservation risk independently of geographic range. The corresponding island-adjusted predicted-probability curves for all seven traits are shown in Supplementary Figure S6.

### 3.6. Triage-ranking performance

Species with lower petal-length variability (CV) were consistently ranked as higher conservation risk across 500 independent cross-validation folds, indicating that this morphological signal is stable and not driven by a single data partition. Discriminatory performance was modest but reproducible (ROC AUC = 0.578, SD = 0.061, 95% CI [0.458, 0.693]; PR AUC = 0.349, SD = 0.060, 95% CI [0.246, 0.481]), with the latter representing a mean lift of 0.075 above the naïve baseline (0.274). Thus, the model identifies Threatened species at a meaningfully higher rate than expected under random assignment of risk scores. A permutation test (B = 10,000) further indicated that this discriminatory capacity exceeds that expected if conservation status were unrelated to floral variability (permutation *P*-value = 0.053; Supplementary Figure S3), supporting a genuine, though modest, biological association rather than chance patterning in the data (Figure 2).

**Figure 2.**
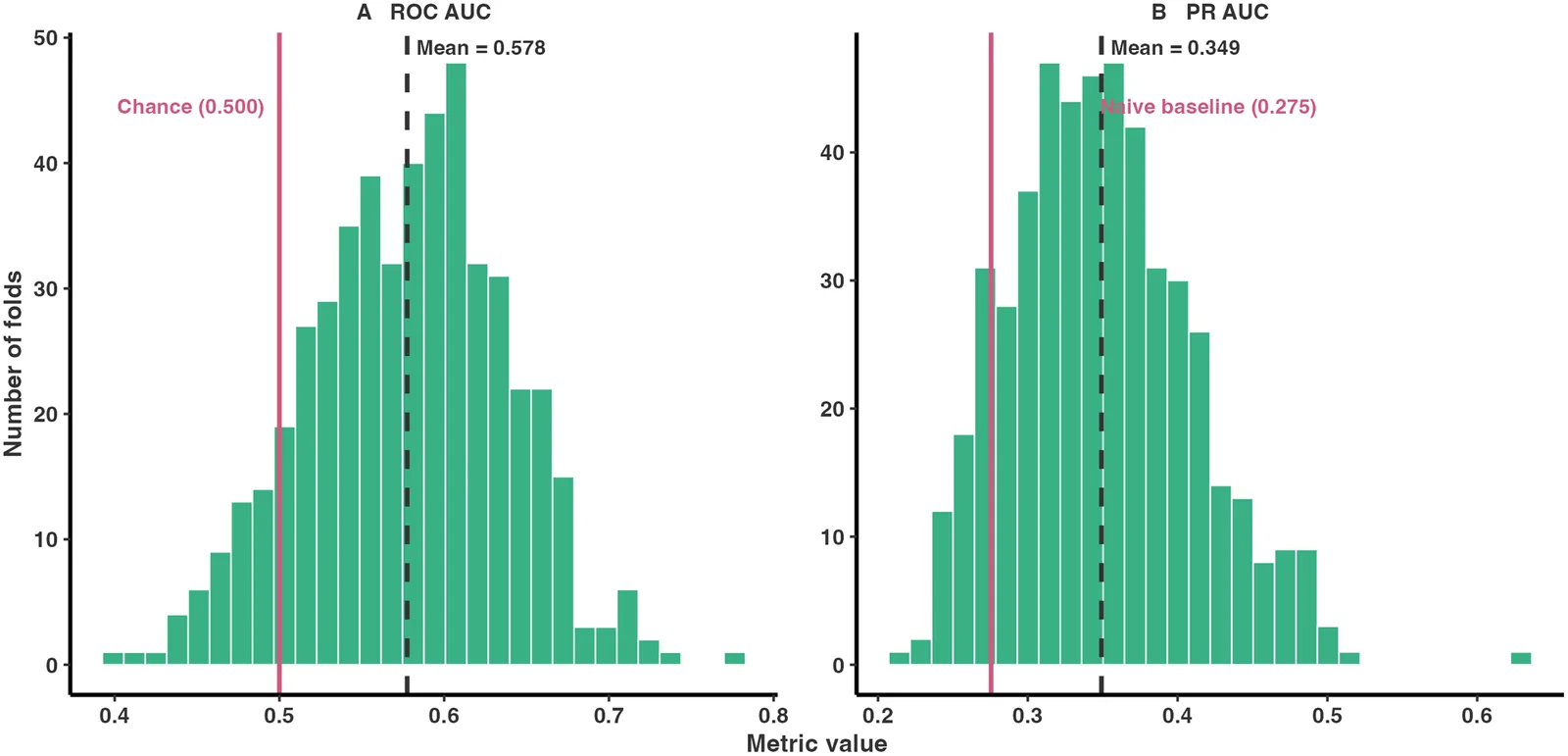
Monte Carlo cross-validation performance distributions for the BIC-averaged floral-CV triage model across 500 stratified 70/30 folds. (A) Distribution of fold-level ROC AUC values. (B) Distribution of fold-level PR AUC values. Dashed vertical line = mean across folds; pink-red vertical line = chance baseline (ROC AUC = 0.500) or naive prevalence baseline (PR AUC = 0.275). For each split, predictions on the heldout test data were generated separately for *m* = 20 multiply imputed datasets (randomly drawn from a pool of *m* = 100 imputations) and then averaged to yield a single prediction per observation before calculating performance metrics. Figure 2. long description.

### 3.7. Threshold-based decision rules for operational triage

The practical question for conservation triage is not whether the model discriminates perfectly, but whether it can reliably flag Threatened species for priority assessment while keeping the evaluation queue manageable. Three decision thresholds address this question differently, each reflecting a different tolerance for missing a genuinely Threatened species (Supplementary Table S1). The cost-sensitive rule (5:1 FN:FP penalty, mean τ = 0.391) is the most precautionary: it correctly identifies 97.3% of Threatened species across folds (mean recall = 0.973, SD = 0.055), meaning that of every 100 genuinely Threatened Caribbean orchids, fewer than three would be overlooked. Fold-to-fold variance is low, indicating that this performance is stable across different subsets of the data. The precautionary screening rule (PR max-F1, mean τ = 0.473) recovers 81.4% of Threatened species (mean recall = 0.814, SD = 0.137) with a smaller false-positive burden, offering a useful intermediate option where assessment resources are more constrained. The balanced rule (Youden’s J, mean τ = 0.506), which treats missed Threatened species and unnecessary assessments as equally costly, recovers 65.3% of Threatened species (mean recall = 0.653, SD = 0.213); high fold-to-fold variance reflects the instability inherent when only 14–20 Threatened species are present per test fold. We recommend the cost-sensitive rule for operational conservation triage.

## 4. Discussion

We find that orchids with greater within-species variability in floral display structures are consistently less likely to be classified as Threatened; species in which individuals are morphologically more uniform are associated with higher conservation risk. This pattern is supported by three independent lines of evidence, BIC model selection, sign-direction analyses and marginal odds ratios, all pointing in the same direction. The fact that the signal is captured by single-trait models rather than trait combinations indicates a parsimonious relationship. We interpret this to mean that the perianth-CV signal is broadly distributed across moderately correlated display traits (all Spearman |rs| ≤ 0.81, variance inflation factor VIF below 5; Supplementary Table S5) rather than reflecting independent biological information about a species’ capacity to buffer environmental and pollinator variability, and no synergistic multi-trait effect is needed to explain the pattern.

The association we report does not arise from shared ancestry. Although threat status is phylogenetically clustered (closely related Caribbean orchids tend to share conservation status, D = 0.62), the link between reduced floral variability and elevated risk persists once we model that clustering. Across ordinary, phylogenetic, and mixed-model frameworks, the trait coefficients retain their sign and magnitude, and two independent methods detect almost no residual phylogenetic signal in risk after floral CV is included. We therefore interpret the perianth-CV signal as a property of the species themselves rather than a by-product of clade membership. The phylogenetic model ranks labellum-length CV marginally ahead of petal-length CV, but we do not read this as identifying a single causal trait: the labellum effect is weak and, assessed univariately, negligible (Table 1). The robust result is instead a broadly distributed, low-magnitude perianth-CV signal, consistent with the modest discrimination reported here.

### 4.1. Petal length and sepal width

Species whose individuals varied more in outer perianth dimensions were less likely to be listed as Threatened (OR < 1), and we interpret this as evidence that within-population morphological variability acts as a form of reproductive insurance. When individuals present a wider range of silhouettes and platform dimensions, the population collectively samples a broader region of pollinator preference space either at the species level or the within species morphological diversity, so that shifts in pollinator identity, abundance or behaviour are less likely to leave the whole population reproductively mismatched. This effect may be amplified in the many orchids pollinated by deception, which offer no reward: because bees and other pollinators learn quickly to avoid unrewarding phenotypes, greater within-population floral variability impedes such avoidance learning and sustains visitation, whereas morphologically uniform (low-CV) populations are more readily learned and avoided, depressing visitation and reproductive success (Gigord et al., 2001). We view this as a variance-buffering, or portfolio, effect: reproductive risk is spread across phenotypes much as bet-hedging spreads it across individuals, so that no single pollinator decline or environmental perturbation drives synchronous failure because some individuals remain functionally matched to prevailing conditions. Greater dispersion in display traits should therefore stabilise population-level reproductive performance under fluctuating plant–pollinator interactions and lower extinction risk even where mean trait values are unchanged (Chevin et al., 2010; Bolnick et al., 2011; Westerband et al., 2021). Reproduction in these orchids is pollination-limited, as it generally is in the family: fruit set is characteristically low and strongly dependent on pollinator service, and reproductive success is highly skewed among individuals and years (Tremblay et al., 2005). Because the conversion of reproductive success into recruitment is itself haphazard and stochastic in orchids, and their populations are subject to rarely-observed demographic transitions, transient dynamics and persistent habitat instability (Tremblay et al., 2021; Ospina-Calderón et al., 2023; Ackerman et al., 2025), the link between floral variability and population persistence is best regarded as probabilistic rather than deterministic.

This interpretation is consistent with observations that orchids generally interact with few, often highly specialised pollinators (Tremblay, 1992; Ackerman et al. 2023), making pollinator–plant-phenotype compatibility a critical component of fitness variation. Importantly, outer perianth traits need not be subject to strong or consistent directional selection to influence extinction risk. Field studies in orchids frequently report weak, spatially variable or temporally inconsistent selection on perianth traits outside the column and labellum. In *Lepanthes rupestris* Stimson, for example, none of the 12 floral characters, including perianth, labellum and column, showed consistent selection coefficients across seven populations based on pollen removal or deposition (Cintrón-Berdecia and Tremblay, 2006).

We interpret these results as evidence that variability in outer perianth traits contributes to demographic resilience through variance buffering and interaction breadth, rather than through trait-specific optimisation. Such traits may function as flexible components of the floral display that enhance robustness to fluctuating pollinator environments, even in the absence of strong, repeatable phenotypic selection.

### 4.2. Column and labellum length

Species exhibiting greater variability in column length were more likely to be classified as Threatened (96% positive BIC weight). The column functions as the primary reproductive interface in orchid flowers, housing both the anther and stigma within a fused structure, and successful pollen transfer depends on precise spatial alignment between the column and specific contact points on the pollinator’s body (Darwin, 1877; Dressler, 1981).

Unlike display traits, where greater variation may expand pollinator compatibility, the column is predicted to operate under stabilising selection. Deviations from optimal column geometry reduce pollination efficiency by preventing correct placement of the pollinium or by misaligning the pollinarium relative to the pollinator’s body (Tremblay and Ackerman, 2001; de Jager and Peakall, 2019). Species pollinated by a single or highly specific pollinator are therefore expected to exhibit lower column CV, whereas species interacting with multiple pollinators may tolerate greater variation.

Consistent with this expectation, column size directly influences pollinarium removal and fruit set. In *L. rupestris*, column dimensions were significant predictors of pollination success (Tremblay, 2006), and the importance of precise column–pollinator fit recurs across diverse orchid systems, including *Habenaria* in South America (Moré et al., 2012), *Cyclopogon* (Benitez-Vieyra et al., 2006) and sexually deceptive Australian genera (de Jager and Peakall, 2019).

Excess within-species dispersion in column dimensions likely desynchronises mechanical fit across individuals within a population. Flowers deviating from the functional range would experience reduced pollinium transfer efficiency. The contrast with display traits is mechanistically coherent: variation in attraction structures may broaden pollinator compatibility, while equivalent variation in reproductive mechanics erodes the precision required for successful pollen transfer.

The labellum-length CV presented an apparent contradiction: the observed median was slightly higher in Threatened species (0.687 vs. 0.569; Wilcoxon *P*-value = 0.858), yet the BIC-weighted sign analysis placed it in a negative direction across every model in which it appeared. Two complementary mechanisms resolve this. The negative regression direction is primarily a suppression artefact. The marginal (univariate) logistic model for labellum-length CV yields a positive coefficient (OR = 1.24), consistent in direction with the other perianth traits. The negative direction appears only when labellum-width CV enters the same model (Spearman r = 0.83 between the two traits): once the variance shared with labellum width is accounted for, the residual variance in labellum length predicts lower risk, reversing the apparent sign. A secondary contributor is non-random missingness. The observed Threatened median rests on only 23 measured values (65.2% missing), compared with 124 Not Threatened species (28.7% missing; chi-square *P*-value <0.001). This differential absence biases the Threatened median upward because only species with adequate measurement records contribute to it. Together, statistical suppression and non-random missing data explain why the descriptive comparison and the regression direction diverge. The resolution is methodological, not biological.

### 4.3. Model performance **and triage application**

The primary finding is biological rather than methodological: orchid species exhibiting lower within-species variability in key floral traits are consistently more likely to be classified as Threatened. Reduced floral coefficients of variation likely reflect the erosion of standing phenotypic variation within populations, precisely the variation that buffers species against environmental change, shifts in pollinator assemblages and demographic bottlenecks. Populations dominated by morphologically uniform individuals offer fewer phenotypic variants upon which selection can act, and when this uniformity extends to traits governing pollinator fit, species may become constrained to a narrow functional niche that becomes maladaptive as conditions change.

The model formalises this biological signal into a continuous risk metric derived entirely from floral measurements available in regional floras (Ackerman, 2014), without requiring demographic surveys or explicit distributional modelling. The fact that this signal remains detectable despite substantial trait missingness (13–54%), CV estimates derived from reported ranges, severe class imbalance and the likelihood that many species are threatened for reasons unrelated to morphology, argues strongly that the association between reduced floral variability and conservation status represents a genuine biological pattern rather than a statistical artefact.

A ROC AUC of 0.578 is modest, but AUC optimises balanced discrimination across all thresholds and is the wrong metric for a triage problem in which the costs of the two error types are asymmetric. Missing a Threatened species is irreversible; a false positive costs only an unnecessary assessment. Weighting false negatives five times more heavily than false positives, the cost-sensitive threshold achieves a mean recall of 0.973 across 500 cross-validation folds (Supplementary Table S1), correctly identifying 97% of Threatened species on average. The permutation test (*P*-value = 0.053) confirms that this performance improves despite the data constraints. For orchid species currently lacking a formal risk assessment, the recommended application is to rank species by predicted probability and direct expedited assessment towards those scoring in the upper quantiles, not to treat scores as determinations of threat status, but to convert a resource-allocation problem into a ranked priority list that existing assessment infrastructure can work through systematically.

### 4.4. Limitations and future directions

The present results are restricted to a small subset of orchids (Caribbean flora). Extension to other geographic regions will require independent regional analyses, given strong regional variation in pollinator assemblages, evolutionary histories and threat drivers. Broadening this framework to additional taxonomic groups (including outside the plant kingdom) will likewise be essential to determine whether the patterns observed here can be generalised beyond orchids. Trait CVs were derived from published trait ranges rather than direct population-level sampling, which introduces estimation error.

Future work should extend this framework to additional regions and clades and incorporate demographic variance alongside morphological variance. For example, CVs of vital rates derived from matrix population models (Hernández-Yáñez et al., 2022) could be used to test whether the petal-length CV signal persists after accounting for phylogenetic structure and uneven sampling effort. In addition, assessing whether floristic datasets accurately represent natural levels of morphological variation in threatened species (Tremblay et al., 2022) would clarify whether reduced trait variance reflects in situ population processes rather than artefacts of data compilation. The approach adopted here is deliberately simple: it summarises floral variability with univariate CVs of linear measurements and does not capture the three-dimensional structure of the flower or the complexity of its shape, curvature and symmetry. These dimensions could be incorporated through geometric morphometrics (Kriebel et al., 2020), which would expose axes of floral variability that linear measurements overlook and would be valuable for understanding how floral disparity arises. For the practical purpose of triaging unassessed species, however, this simplicity is an asset: the CV-based measures used here are computationally light and can be derived directly from published floras, whereas the imaging, landmarking and computation that a full morphometric analysis entails would be difficult to justify at the scale of an entire flora.

### 4.5. Synthesis

A persistent theme in extinction risk research is the search for intrinsic biological attributes that mediate sensitivity to external pressures (Purvis et al., 2000; Brook et al., 2008). Historically, this effort has emphasised mean trait values, particularly life history, demographic and spatial traits, which have proven broadly predictive across taxa (Owens and Bennett, 2000; Cardillo et al., 2005; Slatyer et al., 2013). By contrast, our results align with emerging theory suggesting that persistence depends not only on central tendency but also on how trait variation is distributed within species (Chevin et al., 2010; Westerband et al., 2021). Reduced ITV can constrain compatibility with fluctuating environments and biological interactions, limiting both short-term buffering and longer-term adaptive responses to change (Bolnick et al., 2011).

By focusing on ITV rather than average morphology, we place floral traits within a broader persistence framework that treats variance as a functional property of populations rather than as a residual noise around an adaptive optimum (Violle et al., 2012; Siefert et al., 2015). In orchids, where reproductive success depends on fine-scale interactions with pollinators, even modest differences among individuals may have disproportionate demographic consequences (Nilsson, 1988; Johnson and Steiner, 1997; Ackerman et al. 2023). Species exhibiting narrow floral variability may therefore experience more synchronised reproductive failure under unfavourable conditions, accelerating decline even when external pressures are similar (Chevin et al., 2010; Westerband et al., 2021).

Trait-specific patterns further indicate that the consequences of variability depend on function. Increased dispersion in display traits may enhance resilience by broadening pollinator compatibility and interaction breadth (Faegri and van der Pijl, 1979; Waser et al., 1996; Aragón and Ackerman, 2004), whereas elevated variation in column dimensions may undermine the mechanical precision required for successful pollen transfer (Darwin, 1877; Tremblay and Ackerman, 2001; de Jager and Peakall, 2019). This contrast is mechanistically coherent and suggests that selection on variance, rather than on means, differs fundamentally between attraction structures and floral traits directly involved in reproductive mechanics (Benitez-Vieyra et al., 2006; Moré et al., 2012). The link between reduced morphological variation and elevated extinction risk appears to transcend taxonomic boundaries: Birskis-Barros et al. (2026) demonstrated that Hawaiian honeycreepers (Drepanidinae) with lower within-species variation in beak shape exhibited narrower isotopic niches and were more likely to have gone extinct, providing independent cross-taxon support for this hypothesis.

We also tested directly whether this association is confounded by geographic range, the strongest known correlate of extinction risk. Using the distribution statements in Ackerman (2014), we classified each species as a single-island endemic or as occurring on two or more islands, and refitted the focal models with this rarity covariate. Single-island endemism was, as expected, a powerful predictor of risk, yet the perianth-CV effects were not diminished by the adjustment: they were preserved and strengthened, with petal-length, sepal-width and labellum-length CV all significant once island occupancy was controlled (Supplementary Table S8). Reduced perianth variability therefore predicts conservation risk independently of geographic range, rather than as an incidental correlate of it. Other species-level covariates, such as island area or deforestation, were not available for this flora, so residual confounding with such factors cannot be entirely excluded; nonetheless, the independence of the floral signal from the dominant rarity axis substantially strengthens the inference.

More broadly, these findings argue for incorporating ITV into comparative extinction risk frameworks alongside traditional predictors. As anthropogenic pressures intensify, persistence may depend less on how closely trait means match current conditions than on how much standing variation populations retain to absorb environmental fluctuation and disruption (González-Suárez and Revilla, 2013; Des et al., 2018). Importantly, the morphological variance signal documented here emerges across a flora subject to diverse and largely unquantified threat drivers (including habitat loss, climate change, overcollection and pollinator disruption), suggesting that floral trait dispersion may act as an intrinsic biological attribute modulating extinction risk independently of the specific external pressures acting on any given species. Extinction may be the rule rather than the exception (Barnosky et al., 2011), but accounting for trait distributions rather than trait averages offers a clearer window into why some lineages persist while others fail.

## Supporting information

10.1017/ext.2026.10021.sm001 Tremblay et al. supplementary material Tremblay et al. supplementary material

## Data Availability

Complete R scripts, imputation caches (m = 100), MCCV fold results (N = 500) and model tables and predictions are openly archived, together with the phylogeny, at https://doi.org/10.5281/zenodo.21418755. Raw floral measurements were compiled from a published regional flora (Ackerman, 2014).

## Supplementary material

The supplementary material for this article can be found at http://doi.org/10.1017/ext.2026.10021.

## Long descriptions

### Table 1. Long description

The table contains seven rows of floral traits ordered by Cliff's delta values.

1. Sepal width: C V not threatened 0.278 (139), I Q R 0.185–0.445; C V threatened 0.207 (46), I Q R 0.160–0.372; P 0.056; Cliff's delta minus 0.188.

2. Sepal length: C V not threatened 0.673 (155), I Q R 0.408–1.040; C V threatened 0.568 (55), I Q R 0.368–1.080; P 0.266; Cliff's delta minus 0.101.

3. Petal length: C V not threatened 0.584 (143), I Q R 0.326–1.000; C V threatened 0.509 (42), I Q R 0.304–1.000; P 0.398; Cliff's delta minus 0.086.

4. Labellum length: C V not threatened 0.569 (124), I Q R 0.280–1.120; C V threatened 0.687 (23), I Q R 0.269–1.060; P 0.858; Cliff's delta minus 0.024.

5. Petal width: C V not threatened 0.248 (125), I Q R 0.120–0.489; C V threatened 0.214 (34), I Q R 0.133–0.403; P 0.906; Cliff's delta minus 0.013.

6. Labellum width: C V not threatened 0.422 (92), I Q R 0.224–0.824; C V threatened 0.456 (18), I Q R 0.186–0.878; P 0.948; Cliff's delta minus 0.010.

7. Column length: C V not threatened 0.384 (104), I Q R 0.202–0.652; C V threatened 0.456 (27), I Q R 0.174–0.709; P 0.948; Cliff's delta plus 0.009.

Sample sizes are indicated in parentheses following the C V values. Negative delta values indicate lower C V in Threatened species.

Navigate back to Table 1..

### Table 2. Long description

The candidate model set within delta-BIC < = 2 from complete-subset selection over 127 models, pooled across 100 imputations. Three single-predictor models qualify: rank 1, petal-length CV, BIC = 298.87, delta-BIC = 0.00, BIC weight 0.203 (normalised 0.509); rank 2, sepal-width CV, delta-BIC = 1.34, weight 0.104 (0.260); rank 3, labellum-length CV, delta-BIC = 1.58, weight 0.092 (0.231). All three are single-feature models.

Navigate back to Table 2..

### Table 3. Long description

For each of seven floral CV traits the table gives the summed BIC inclusion weight, the percentage of models in which the coefficient was negative versus positive, the marginal univariate odds ratio (OR) with 95% CI, and a direction label. Five perianth traits are predominantly negative (OR % 1, lower CV -> higher risk): sepal width (weight 0.348; 100% negative; OR 0.326 [0.077, 1.391]), petal length (0.516; 100%; OR 0.597 [0.349, 1.023]), labellum length (0.267; ~100%; OR 0.633 [0.352, 1.139]), plus sepal length (mixed, 57% negative) and labellum width (78% negative). Petal width is mostly positive (82%). Column length is robustly positive (96% positive; OR 0.954 [0.417, 2.183]; higher CV -> higher risk), consistent with the mechanical-precision hypothesis. All confidence intervals include 1.

Navigate back to Table 3..

### Figure 2. Long description

A two-panel figure with a shared y-axis labeled Number of folds ranging from 0 to 50 and a shared x-axis labeled Metric value.

Panel A, titled R O C A U C, displays a green histogram of metric values ranging from approximately 0.4 to 0.8. A solid pink vertical line at 0.500 is labeled Chance 0.500. A dashed black vertical line marks the Mean at 0.578. The distribution is roughly bell-shaped, peaking between 0.55 and 0.65.

Panel B, titled P R A U C, displays a green histogram of metric values ranging from approximately 0.2 to 0.65. A solid pink vertical line at 0.275 is labeled Naive baseline 0.275. A dashed black vertical line marks the Mean at 0.349. The distribution is skewed slightly to the right, with the highest concentration of folds between 0.3 and 0.4.

Navigate back to Figure 2..

### Figure 1. Long description

A multi-panel figure containing seven line graphs arranged in two rows. The top row has four panels and the bottom row has three. All panels share a common Y-axis labeled Predicted probability of being Threatened, ranging from 0% to 60%, and a common X-axis labeled Coefficient of variation (C V).

Each panel includes a horizontal dashed line at 27.5% representing the observed prevalence. A solid teal line represents the mean predicted probability, surrounded by a light teal shaded 95% multiple imputation envelope.

Top Row (Left to Right):

* Sepal W (n = 127): Shows a steep exponential decay curve starting near 35% and dropping toward 0% as C V increases. Down arrow, p = 0.129 [N S].

* Petal L (n = 135): Shows a steady downward curve starting near 35% and ending near 5%. Down arrow, p = 0.060 [dagger symbol].

* Labellum L (n = 103): Shows a gradual downward curve starting near 35% and ending near 10%. Down arrow, p = 0.126 [N S].

* Sepal L (n = 146): Shows a gradual downward curve starting near 33% and ending near 10%. Down arrow, p = 0.169 [N S].

Bottom Row (Left to Right):

* Labellum W (n = 78): Shows a shallow downward linear trend starting at 30% and ending near 18%. Down arrow, p = 0.348 [N S].

* Petal W (n = 113): Shows a very shallow downward trend, nearly flat, with a widening confidence ribbon. Down arrow, p = 0.714 [N S].

* Column L (n = 91): Shows a nearly horizontal flat line at the 27.5% threshold with a symmetrical widening ribbon. Down arrow, p = 0.911 [N S].

Navigate back to Figure 1..
