# Supplementary material for: Inherently at risk: Low floral morphological variability predicts conservation risk
Source: Camb Prism Extinct. 2026 Sep 29;4:e15. doi: 10.1017/ext.2026.10021 (PMC13748988; doi:10.1017/ext.2026.10021)
Supplement: Supplementary file 1 [file S2755095826100217sup001.docx]

**Supplementary Material**

***Inherently at Risk: Low morphological variability predicts conservation risk***

**Tremblay RL, Leon-Rodriguez A, Colon-Miranda JD, Melendez Hebib V.**

This supplement contains six figures and nine tables providing additional analytical detail supporting the results reported in the main text. Figure S1 and Figure S2 expand on the model selection and inference results. Figure S3 and Table S1 present a Monte Carlo cross-validation (MCCV) robustness assessment conducted independently of the primary held-out test set evaluation reported in the main text. The two evaluations are complementary: the primary evaluation (main text) provides a single unbiased estimate of out-of-sample performance on a stratified held-out test set (*n* = 73; ROC AUC = 0.551, PR AUC = 0.334); the MCCV assessment (this supplement) evaluates the stability of that performance estimate across 500 repeated train–test splits, addressing whether the primary result depends on a particular random partition of the data. Tables S2–S4 report additional analyses assessing and controlling for phylogenetic non-independence.

**Figure S1.** BIC-weighted variable inclusion probabilities for all seven floral coefficient-of-variation (CV) traits across the complete set of 127 candidate logistic regression models for Threatened status, pooled across *m* = 100 imputed datasets. The summed BIC weight for each trait reflects its cumulative support across the model space, weighted by relative BIC evidence. The dashed vertical line marks the uniform inclusion weight (*w* = 1/7 ≈ 0.143), below which a trait contributes less support than expected under an uninformative prior over candidate models.

**Figure S2.** Marginal odds ratios for the predicted probability of Threatened status per unit increase in within-species floral CV for each of the seven traits, estimated from univariate logistic regressions pooled across *m* = 100 imputed datasets using Rubin's rules. Error bars show 95% confidence intervals on the log scale. Traits are ordered from smallest to largest OR.

**Figure S3.** Permutation null distribution for mean ROC AUC from the MCCV robustness assessment, based on *B* = 10,000 permutations of the outcome labels, each evaluated over 20 Monte Carlo cross-validation folds. The observed mean ROC AUC across 500 MCCV folds (0.578) is shown as a vertical line; the one-sided permutation *P*-value is the proportion of permuted mean AUCs equal to or exceeding the observed value (*P*-value = 0.053). The modest but consistent discrimination observed across folds, paired with the permutation result falling just outside conventional significance, corroborates the primary held-out test set result (ROC AUC = 0.551) and confirms that the signal is not an artefact of a single data partition.

Table S1. Threshold-based classification performance for three decision rules evaluated across the 500 MCCV folds using stratified 70/30 train–test splits (approximately 20 Threatened species per test fold). Metrics are reported as mean (SD) across folds. For each rule, τ is the probability threshold applied to the model's risk score; recall (sensitivity) is the proportion of truly Threatened species flagged, precision the proportion of flagged species that are Threatened, specificity the proportion of Not-Threatened species correctly not flagged, and F1 the harmonic mean of precision and recall. Performance estimates from repeated cross-validation are somewhat more optimistic than those from the single held-out test set due to the smaller and more variable test folds inherent in the MCCV design; the held-out test set results should be treated as the primary performance estimate. Nevertheless, the directional conclusions are consistent across both evaluations: the cost-sensitive rule (5:1 false-negative:false-positive penalty) consistently achieves the highest sensitivity across MCCV folds (mean = 0.973, SD = 0.055), and the ranking of decision rules by recall is identical between the MCCV and held-out assessments.

| **Decision rule** | **Mean τ** | **Recall (± SD)** | **F1 (± SD)** | **Precision** | **Specificity** |
| --- | --- | --- | --- | --- | --- |
| Balanced (Youden’s J) | 0.506 | 0.653 ± 0.213 | 0.460 ± 0.062 | 0.394 | 0.571 |
| Precautionary screen (PR max-F1) | 0.473 | 0.814 ± 0.137 | 0.477 ± 0.037 | 0.347 | 0.387 |
| Cost-sensitive  (5:1 FN:FP) | 0.391 | 0.973 ± 0.055 | 0.468 ± 0.029 | 0.310 | 0.166 |

**Phylogenetic non-independence analyses**

To determine whether the trait-risk associations reflect phylogenetic non-independence, we obtained a phylogeny of the study species from the Open Tree of Life synthetic tree (233 of 240 species placed) with Grafen branch lengths, tested for phylogenetic signal in threat status (Fritz and Purvis D), repeated the full 127-model BIC selection under phylogenetic logistic regression (phyloglm), and refitted the focal models under genus- and phylogenetic-random-effect mixed models. Threat status showed significant phylogenetic clustering (Table S2), but the trait effects were robust to correction: the ΔBIC ≤ 2 set retained three single-trait perianth-CV models (Table S3) and coefficient signs and magnitudes were stable across all frameworks (Table S4). The estimated residual phylogenetic correlation was negligible (phyloglm α ≈ 54, near its upper bound; phylogenetic random-effect variance ≈ 0), indicating that little phylogenetic structure in risk remains once floral CV is included.

Table S2. Phylogenetic signal in threat status (Fritz and Purvis D). D = 0 indicates Brownian-threshold clumping and D = 1 phylogenetic randomness; both trees reject a random distribution of threat status.

| **Tree** | **Tips** | **D** | **P (random)** | **P (Brownian)** |
| --- | --- | --- | --- | --- |
| Open Tree of Life | 233 | 0.62 | < 0.001 | < 0.001 |
| Taxonomy (genus) | 240 | 0.86 | 0.016 | < 0.001 |

Table S3. Best-supported model set (ΔBIC ≤ 2): non-phylogenetic versus phylogenetic selection. Models are single floral coefficient-of-variation (CV) predictors, and the phylogenetic selection uses phylogenetic logistic regression (phyloglm). BIC is not comparable across the two approaches (different likelihood scale and n); only within-approach ranks and ΔBIC are meaningful.

| **Model (single-trait CV)** | **Non-phylo rank (ΔBIC)** | **Phylo rank (ΔBIC)** |
| --- | --- | --- |
| Labellum-length CV | 3 (1.58) | 1 (0.00) |
| Petal-length CV | 1 (0.00) | 2 (1.65) |
| Sepal-length CV | 6 (3.92) | 3 (1.85) |
| Sepal-width CV | 2 (1.34) | 5 (2.54) |

Table S4. Cross-framework coefficient comparison for the focal single-trait models, pooled across m = 100 imputations (β on the logit scale, *P*-value in parentheses). Perianth traits are negative in every framework; column-length CV is near zero as a single predictor and positive only in multi-trait models (n/a = not fitted separately). The four frameworks are phylogenetic logistic regression (phyloglm), a class-weighted genus-random-effect generalized linear mixed model (Genus GLMM), and unweighted mixed models with a phylogenetic (Phylo-RE) or genus (Genus-RE) random effect.

| **Trait** | **phyloglm** | **Genus GLMM (wt.)** | **Phylo-RE GLMM** | **Genus-RE GLMM** |
| --- | --- | --- | --- | --- |
| Petal-length CV | −0.74 (0.082) | −0.58 (0.19) | −0.47 (0.16) | −0.46 (0.18) |
| Sepal-width CV | −1.96 (0.054) | −1.70 (0.23) | −0.88 (0.35) | −1.00 (0.31) |
| Labellum-length CV | −0.54 (0.12) | −0.54 (0.24) | −0.35 (0.33) | −0.43 (0.25) |
| Column-length CV | −0.24 (0.75) | n/a | +0.25 (0.65) | +0.07 (0.89) |

Genus random-effect variance (unweighted) ≈ 0.6 (intraclass correlation ≈ 0.15); phylogenetic random-effect variance ≈ 0; phyloglm α ≈ 54. A phylogeny of threat status can be produced from the archived tree using the accompanying R script, archived at https://doi.org/10.5281/zenodo.21418755.

**Collinearity among predictors**

Table S5. Pooled Spearman rank correlation matrix among the seven floral CV predictors (mean across m = 100 imputations; lower triangle shown). Spearman is used because the CVs are strongly right-skewed and non-normal (skewness +1.5 to +6.2; all Shapiro-Wilk *P*-values < 0.001). Collinearity is moderate: all pairwise |rs| ≤ 0.81, and all variance inflation factors are below 5, from the fitted linear models (sepal width 1.73, sepal length 3.02, petal width 1.69, petal length 4.04, labellum length 4.79, labellum width 3.05, column length 2.14; maximum 4.79) and from rank-transformed predictors (maximum 4.80), so no predictor was excluded by the |r| ≥ 0.90 filter.

|  | **Sepal w** | **Sepal l** | **Petal w** | **Petal l** | **Lab. l** | **Lab. w** | **Col. l** |
| --- | --- | --- | --- | --- | --- | --- | --- |
| **Sepal width** | 1.00 |  |  |  |  |  |  |
| **Sepal length** | 0.56 | 1.00 |  |  |  |  |  |
| **Petal width** | 0.56 | 0.36 | 1.00 |  |  |  |  |
| **Petal length** | 0.58 | 0.81 | 0.49 | 1.00 |  |  |  |
| **Labellum length** | 0.55 | 0.57 | 0.55 | 0.75 | 1.00 |  |  |
| **Labellum width** | 0.47 | 0.44 | 0.56 | 0.61 | 0.80 | 1.00 |  |
| **Column length** | 0.55 | 0.59 | 0.54 | 0.65 | 0.62 | 0.66 | 1.00 |

Figure S4. Spearman rank correlation heatmap among the seven floral CV predictors (pooled across m = 100 imputations; lower triangle). Warmer colours indicate stronger positive correlation. All pairwise |rs| ≤ 0.81 and all variance inflation factors are below 5, indicating moderate but not severe collinearity.

**Missingness and imputation robustness**

Table S6. Missingness by conservation status for the seven floral CV predictors (all 240 species; percentages correspond to the Table 1 sample sizes). Missingness is higher among Threatened species for five of seven traits (χ² *P*-value < 0.05), reflecting that rarer, threatened orchids are less completely described in the source flora. This outcome-related missingness, combined with excluding the outcome from the imputation model (to avoid target leakage), renders the trait–risk estimates conservative.

| **Trait** | **% missing** | **% in Threatened** | **% in Not Threatened** | **χ² *P*** |
| --- | --- | --- | --- | --- |
| **Sepal width** | 22.9 | 30.3 | 20.1 | 0.13 |
| **Sepal length** | 12.5 | 16.7 | 10.9 | 0.33 |
| **Petal width** | 33.8 | 48.5 | 28.2 | 0.005 |
| **Petal length** | 22.9 | 36.4 | 17.8 | 0.004 |
| **Labellum length** | 38.8 | 65.2 | 28.7 | < 0.001 |
| **Labellum width** | 54.2 | 72.7 | 47.1 | 0.001 |
| **Column length** | 45.4 | 59.1 | 40.2 | 0.013 |

Table S7. Marginal odds ratios (OR, with *P*-value in parentheses) for each focal trait under three imputation strategies (training set; class-weighted logistic regression pooled by Rubin's rules): the primary analysis (MICE excluding the outcome), a re-imputation including the outcome, and complete-case analysis. OR < 1 (lower CV → higher risk) in every cell, so the direction is robust to the imputation model; the outcome-included estimates are as strong or stronger, confirming that excluding the outcome makes the primary estimates conservative.

| **Trait** | **Primary (outcome-excluded)** | **Outcome-included** | **Complete-case** |
| --- | --- | --- | --- |
| **Petal-length CV** | 0.597 (0.058) | 0.565 (0.041) | 0.635 (0.089; n = 135) |
| **Sepal-width CV** | 0.326 (0.126) | 0.281 (0.088) | 0.320 (0.131; n = 127) |
| **Labellum-length CV** | 0.633 (0.122) | 0.639 (0.181) | 0.708 (0.295; n = 103) |

Figure S5. Observed (blue) versus imputed (red) value distributions for the seven floral CV predictors across the m = 100 imputations of the training set. The imputed distributions closely track the observed distributions, indicating the imputations are plausible.

**Geographic covariate: island endemism**

Table S8. Focal-trait odds ratios (OR, with *P*-value in parentheses) for conservation risk, with and without adjustment for single-island endemism, pooled across m = 100 imputations (class-weighted logistic, Rubin's rules; n = 154 training species with distribution data from Ackerman 2014). Single-island endemism is a strong predictor of risk (OR 23 to 33), yet every perianth-trait CV effect is preserved or strengthened after adjustment and becomes significant, whereas column-length CV remains near unity. Reduced perianth-CV therefore predicts risk independently of geographic range.

| **Trait** | **CV alone: OR (p)** | **CV + island: OR (p)** | **Island: OR (p)** |
| --- | --- | --- | --- |
| **Petal-length CV** | 0.607 (0.046) | 0.334 (0.017) | 32.8 (< 0.001) |
| **Sepal-width CV** | 0.549 (0.105) | 0.392 (0.042) | 27.3 (< 0.001) |
| **Labellum-length CV** | 0.690 (0.099) | 0.444 (0.021) | 32.8 (< 0.001) |
| **Column-length CV** | 0.900 (0.590) | 0.869 (0.586) | 23.2 (< 0.001) |

**Figure S6.** Predicted probability of being Threatened as a function of within-species floral coefficient of variation (CV) for all seven floral traits, by geographic range class, from additive logistic models (CV + single-island endemism) fitted to the training-set imputations. Pink curves denote single-island endemics and teal curves species occurring on two or more islands; shaded bands are 95% envelopes across the m = 100 imputations and the dashed line marks overall Threatened prevalence. The model is additive, a shared CV slope with a group-specific intercept, rather than an interaction: single-island endemism raises baseline risk while reduced perianth-CV continues to predict risk within each range class, consistent with Table S8. The near-flat appearance of the widespread (teal) curves is largely a floor-compression effect of the probability scale under a shared slope, not an absence of a CV effect. Predicted probabilities are unweighted; per-panel n reflects trait-specific completeness among training-set species with distribution data.

**Table S9.** Targeted interaction models for conservation risk in Caribbean orchids (*n* = 240 species, of which 66 are Threatened). Following a reviewer's suggestion, we tested whether interactions between the leading floral traits improve on the single-trait models used in the main analysis. The response is binary conservation status (Threatened versus Not Threatened), and the predictors are species-level coefficients of variation (CV, a scale-free measure of within-species dispersion) of the three most-supported floral traits: petal length, sepal width, and labellum length. Each model is a class-weighted logistic regression (inverse-frequency observation weights offset the class imbalance) fitted to each of *m* = 100 multiply-imputed training datasets; the Bayesian Information Criterion (BIC, for which lower values indicate better support and a difference of about two units is conventionally treated as meaningful) was computed within each imputation and averaged across them. Predictors were mean-centred before forming products, which leaves BIC unchanged. 'Single' models contain one CV, 'additive' contains two CVs without their product, and 'interaction' adds the product term. ΔBIC is the increase in BIC relative to the best single-trait model (petal-length CV, BIC = 298.87); larger positive values indicate a poorer model. The interaction term is the Rubin's-rules pooled odds ratio (OR) for the product of the two mean-centred CVs, with its 95% confidence interval (CI) and *P*-value; an OR near 1, or a CI spanning 1, indicates no interactive effect. Every interaction raises BIC by roughly eight units (ΔBIC > 2) with a non-significant product term (all *P*-values > 0.5), and even the additive two-trait model is poorer than the single petal-length model. There is therefore no support for an interaction among the leading floral traits, and the single-trait hierarchy reported in the main text is not an artefact of unmodelled interactions.

| **Model** | **BIC** | **ΔBIC** | **Interaction term (OR, 95% CI, *P*-value)** |
| --- | --- | --- | --- |
| Petal-length CV (single) | 298.87 | 0.00 | n/a |
| Sepal-width CV (single) | 300.21 | 1.34 | n/a |
| Labellum-length CV (single) | 300.45 | 1.58 | n/a |
| Petal-length + Sepal-width (additive) | 302.11 | 3.24 | n/a |
| Petal-length × Sepal-width (interaction) | 307.01 | 8.14 | OR = 1.16 [0.46, 2.88], *P*-value = 0.76 |
| Petal-length × Labellum-length (interaction) | 307.23 | 8.36 | OR = 0.84 [0.43, 1.66], *P*-value = 0.62 |
| Sepal-width × Labellum-length (interaction) | 306.64 | 7.77 | OR = 0.42 [0.02, 10.84], *P*-value = 0.60 |
