# Supplementary material for: Inherently at risk: Low floral morphological variability predicts conservation risk
Source: Camb Prism Extinct. 2026 Sep 29;4:e15. doi: 10.1017/ext.2026.10021 (PMC13748988; doi:10.1017/ext.2026.10021)
Supplement: Peer review history

# Reviewer report: Review: Inherently at risk: Low floral morphological variability predicts conservation risk — R0/PR2

**DOI:** 10.1017/ext.2026.10021.pr2

**Authors:** Melissa Whitman

Review of “Inherently at Risk: Low floral morphological variability predicts conservation risk” for Cambridge Prisms: Extinction by Tremblay et al. 2026

Overall

The research, specifically on how low intraspecific trait variability is associated with conservation prioritization, fits the scope of the journal Cambridge Prisms: Extinction. Orchids have disproportionate risk of extinction due to rarity and specialized ecological relationships, and this manuscript provides a framework on how to identify which species are most at risk. The authors use three different methods (class-weighted logistic regression w/ model selection, sign direction analyses, and a held-out test set using AUC sensitivity of predictions for identifying threatened species), plus the inclusion of multiple imputation for missing data, with the unified finding that that lower CV is equated with higher risk. My primary recommendation is for the authors to rephrase parts of the manuscript so that a more active voice is used, with clear identification of knowledge gained from this study, their interpretations of the findings, and recommendations. Methods used in the manuscript are of broader appeal, not just applicable to the study organisms or region.

Impact Statement

The introduction clearly states the knowledge gap, being the focus of many studies on trait averages vs the amount of intraspecific trait variance, and how this may influence extinction risk. Possible changes would be to identify the study system (Why this location? Is it a knowledge rich area? High conservation priority? Other?), followed by how it connects to broader appeal of the study (i.e. ending statement).

Line 12 & 15. Multiple sentences starting with “This”. Reword to be more specific and to be less redundant, consider more active in phrasing e.g. “Our study provides…”.

Line 23. Reword to more active voice, take ownership of the framework you developed.

Abstract

The abstract is well written and within the word count. However, as it is now, it is more methods/results heavy in presentation (which may not be wrong depending on journal scope); consider cutting or condensing the sign direction analyses and ROC AUC section, with less details about the specific numbers, to make space for another sentence about the interpretation of the findings. For instance, I do not yet know how many threatened species were evaluated or general assessment of extinction risk for the clade/region. Similarly, more on theory of why reduced intraspecific variation matters could be expanded on. The inclusion of a second abstract in Spanish broadens the readership interest and potential application of the study findings.

Introduction

Consider adding additional sentences at the end stating overall hypothesis and specific predictions (in a more specific manner than shown), including if any a priori expectations on which traits may matter most (e.g. did you expect a difference in variance importance for pollinator attracting vs receiving traits)?

Line 6. Specify what is responding (e.g. species’ or simply “the” to “their” to link back to lineage)

Line 27. Consider adding the “classic” paper on moth pigmentation by Kettlewell

Materials and Methods

The methods used are sound and clearly stated. The exhaustive approach of 127 (singular or additive) models is not everyone’s preference, but that is a debate beyond this paper. One question, did the authors consider targeted interaction models also? Adding all possible interactions would not have worked due to computations involved, but a few targeted interaction models might have been informative.

Line 57. Do not indent first paragraph after subheading (but check journal format guidelines).

Line 59. Include units (I assume mm).

Line 62. Assumption of normal distribution of traits, but was this confirmed as a part of general trait quality control prior to analyses?

Line 65. Include brief note about how many genera or major clades, etc… geographical range of species if known, such as note on proportion of species that are endemic, threatened, cosmopolitan.

Line 67. Include number of species excluded (was it roughly a third of the diversity?)

Line 71 (and beyond). Italicize n and other statistical abbreviations

Line 77. Brief statement as to why and what kind of missing (e.g. Missing At Random, MAR); see “Missing data in ecology: Syntheses, clarifications, and considerations” by Dumelle et al. 2025 in Ecological Monographs.

Line 78. Site package or method used, if applicable.

Line 81. Define BIC first before abbreviation, cite Schwarz, G. (1978) Annals of Statistics; ditto for AICc (maybe add note about usage for small sample sizes, how conservative BIC is vs AICc for model selection).

Line 87. Pooled via Rubin’s rules? If so, cite. Note, found on Line 98. Ignore or move sentences.

Line 103. Add space (n = 240)

Line 108. Include full name before abbreviating to avoid confusion (e.g. PR AUC = Precision-Recall Area Under the Curve), possibly add what the difference is between ROC vs PR approach (or reword line 110 slightly)

Line 135. The cross-validation slash is oddly high, check font.

Line 144. Add what R version (e.g. 4.5.3) and update to 2026 unless using old version.

Line 144. Change to “mice” or small caps font to differentiate name of package

Line 148. Available where? Zenodo? Dryad? Personal communication? Possibly combine with following sentence for clarification.

Results

The results are clearly stated and supported.

Line 166. Consider changing p = 0.056 to P-value = 0.056

Line 180. Use italics consistently throughout paper

Line 184. Consider a post-hoc model with top traits examined (e.g. petal length * sepal width) to confirm hierarchy.

Line 240. If P-value is marginal, consider increasing the number of permutations to 10k.

Discussion

My main criticism with the discussion is that the overall voice/style is too passive. Revise to add a sense of clear acknowledgement of your own interpretations, speculations, and how specifically this study enhances overall knowledge of the subject and hypotheses explored.

Line 264. Less passive voice, own the fact that the observed pattern (i.e. uniformity = risk) is supported by your findings.

Line 267. Use of “suggests” in two adjacent sentences. Change “This suggests” to something stronger, such as “Our interpretation is that individual display structures are [specific mechanism] because of x, y, z”

Line 272. This paragraph reads as a bit too succinct and passive; consider rewording for conceptual depth.

Line 280. No, it is your interpretation. Reword to avoid starting with “This” and linkage to your prior findings.

Line 285. Possibly add taxonomic authority after name.

Line 308. Abbreviate to L. rupestris since previously stated and no other species from genera starting with the same letter.

Line 337. In my opinion, it is better to avoid the dreaded em dash, since it is increasingly associated with AI writing styles.

Line 370. Consider also expanding the morphological framework to include morphometric analyses where the complexity of flower shape, curvature, and symmetry is incorporated. See work by Ricardo Kriebel, such as “Pollinator shifts, contingent evolution, and evolutionary constraint drive floral disparity in Salvia (Lamiaceae): evidence from morphometrics and phylogenetic comparative methods" in Evolution.

# Reviewer report: Review: Inherently at risk: Low floral morphological variability predicts conservation risk — R0/PR3

**DOI:** 10.1017/ext.2026.10021.pr3

This ms describes an analysis of the statistical associations between species-level extinction risk and intraspecific phenotypic variation, using variation in floral characters in a clade of Caribbean orchids as a case study. The ms is well written and clearly organized, the data seems to be of high quality, and the analysis seems thorough and carefully thought through (with some exceptions detailed below). My questions and comments are as follows:

Abstract: I’m not sure its really necessary to quote test statistics in the abstract. Probably better to simply give a verbal description of the results.

Introduction: the authors do a good job of providing context for the analysis in terms of the broader issues of trait variation and how it might play into extinction vulnerability, and then giving a good rationale for the appropriateness of the study system. However, I feel that the rationale for the study needs to be more firmly grounded in conservation decision making. There are hundreds of papers in the literature that present tests showing that extinction risk is associated with some traits or other, and assuming that this is self-evidently important for conservation planning. I think the authors need to put in a bit more effort to connect their findings with real-world conservation decisions, beyond the current vague allusions to triage and ranking species according to trait variability. How is conservation planning in the Caribbean really done? Do planners really sit down with lists of species ranked in some way, then simply direct funding to the ones at the top? Or is there a complex set of costs, benefits, opportunities, competing interests, political and social constraints, etc, that they need to weigh up when allocating funding to projects? I understand that the authors might see their study as a purely scientific one and not want to get into the field of conservation decisions, but they should consider whether science that aims to support conservation can really be isolated from on the ground decision making. A possible alternative option might be to remove any claims that the work can directly inform conservation planning, and reframe it as a contribution to simply understanding the ecology of species decline.

Line 65: what is the advantage of collapsing IUCN categories to a binary response? You lose a lot of valuable information this way.

Linde 67: I’d like to see more discussion of the implications of ignoring Data Deficient / Not Evaluated species. Firstly, what proportion of species are these? Leaving them out is probably not simply a neutral step, because DD/NE species are very often more threatened than evaluated species.

Statistical modelling: the modelling approach seems thorough, but it doesn’t account in any way for phylogenetic non-independence. One thing that has become clear from the large literature on correlates of threat status is that threat status almost always shows phylogenetic signal, and so there is the potential for tests to suffer from pseudoreplication and give misleading results. I know that classification/machine learning type tests don’t make the same assumptions about independence of errors that linear models do, but to my mind that doesn’t make these methods immune from phylogenetic non-independence. Imagine, for example, if 95% of threatened species were in one genus, and there were factors other than floral traits that were unique to that genus, that may also influence extinction risk?

This raises another important analytical issue, which is that there were no covariates included in the models, ie the only predictors tested were those that were the focus of the hypothesis. So there is no way of knowing whether the statistical associations with floral variation were not the incidental result of covariation with other traits, or for that matter with geographic factors (e.g. whether a species is found on an island or continent, how large or heavily deforested the islands are, etc. The models really need to be expanded to include this kind of information if you want to draw robust conclusions about the influence of floral traits.

Line 76: 57-73% seems like a very high rate of missingness – can multiple imputation really be trusted with these levels? Please provide some more information that gives confidence in the imputed data.

Line 130: why include a threshold that treats false negative and positives as equally costly, when you have just explained in the previous paragraph that false negatives are more costly in a conservation context?

Line 148: Not sure what CPE’s requirements are for data availability but best practice is to make the code and data freely available on a repository like Zenodo or GitHub.

Line 160-161: It seems likely to me that the different floral traits will show some degree of collinearity. Was this tested for, and can you really conclude that each predictor can be considered to have an independent influence on extinction risk? The single-trait models were better fits to the data than multi-predictor models, but this might not be unexpected if the predictors were all collinear. I’d like to see a correlation matrix among predictors, variance inflation factors, or something that demonstrates that treating predictors independently is justified. I think that this is important given that the authors draw a biological conclusion from this at lines 267-268.

Line 276-277: this would seem to be a fair conclusion to draw if there was evidence that pollination rates are limiting recruitment and that this is contributing to low population growth – is there any such evidence?

# Editor report: Recommendation: Inherently at risk: Low floral morphological variability predicts conservation risk — R0/PR4

**DOI:** 10.1017/ext.2026.10021.pr4

**Affiliations:** Durrell Institute of Conservation and Ecology, University of Kent, United Kingdom of Great Britain and Northern Ireland

This manuscript looks at the interesting question of morphological variation and extinction risk. Having read the manuscript and the reviewers comments, I do agree that there may be an issue of phylogenetic non-independence and therefore it would be good if this could be taken into consideration in the revision. I look forward to seeing the revised manuscript.

# Editor report: Decision: Inherently at risk: Low floral morphological variability predicts conservation risk — R0/PR5

**DOI:** 10.1017/ext.2026.10021.pr5

**Affiliations:** Biological Sciences, The University of Queensland Faculty of Science, Australia

No accompanying comment.

# Reviewer report: Review: Inherently at risk: Low floral morphological variability predicts conservation risk — R1/PR7

**DOI:** 10.1017/ext.2026.10021.pr7

**Authors:** Melissa Whitman

The overall paper has improved as compared to the first version. The authors have specifically met my primary revision recommendations, namely the rewording of the manuscript using a more active voice. Other improvements that have been implemented include the addition of more references related to historic studies of species' traits and statistical methods, as well as increasing the number of imputations when P-values were marginal. The one minor change that needs to be addressed is for consistent use of italics, specifically for all unit abbreviations (e.g. n for sample size), statistical packages (e.g. ggplot, in italics or whatever the journal requires), and the term a priori. Another possible improvement, which is purely optional in my opinion, would be to replicate the number of imputations from 100 to 1000, with further pooling of results using Rubin’s rules, and to check if that larger unit of replication changes the outcome of any analyses (i.e. robustness check and/or possible shift in marginal results), however this can be more computationally intensive, possibly requiring additional effort and time with regards to revisions.

# Reviewer report: Review: Inherently at risk: Low floral morphological variability predicts conservation risk — R1/PR8

**DOI:** 10.1017/ext.2026.10021.pr8

The authors have done a very good job of addressing my original concerns through re-analysis and genuine engagement with issues such as the conservation decision context for the study, phylogenetic non-independence, collinearity etc. The study is now far more solid and robust. Just one comment, its not really clear what is meant by “A transparent screen computable from data already 179 held in published floras” at line 178.

# Editor report: Recommendation: Inherently at risk: Low floral morphological variability predicts conservation risk — R1/PR9

**DOI:** 10.1017/ext.2026.10021.pr9

**Affiliations:** Durrell Institute of Conservation and Ecology, University of Kent, United Kingdom of Great Britain and Northern Ireland

Thanks you for your fantastic job of revising your manuscript. As you can see the reviewers appreciated your efforts and have recommended acceptance. They both raise a couple of very minor issue that can be picked up in the proof stage namely consistency of using italics and slightly rewording one sentence. Many thanks again for submitting this fascinating manuscript and look forward to seeing it published.

# Editor report: Decision: Inherently at risk: Low floral morphological variability predicts conservation risk — R1/PR10

**DOI:** 10.1017/ext.2026.10021.pr10

**Affiliations:** Durrell Institute of Conservation and Ecology, University of Kent, United Kingdom of Great Britain and Northern Ireland

No accompanying comment.
